# Partners in Postmortem Interval Estimation: X-ray Diffraction and Fourier Transform Spectroscopy

**DOI:** 10.3390/ijms24076793

**Published:** 2023-04-05

**Authors:** Leticia Rubio, Juan Suárez, Stella Martin-de-las-Heras, Sara C. Zapico

**Affiliations:** 1 Departamento de Anatomía Humana, Medicina Legal e Historia de la Ciencia, Facultad de Medicina, Universidad de Málaga, 29071 Málaga, Spain; juan.suarez@uma.es; 2 Department of Chemistry and Environmental Sciences, New Jersey Institute of Technology, Tiernan Hall 365, Newark, NJ 07102, USA; sc338@njit.edu; 3 Instituto de Investigación Biomédica de Málaga-IBIMA, 29590 Málaga, Spain; 4 Department of Anthropology, NMNH-MRC 112, Smithsonian Institution, Washington, DC 20560, USA

**Keywords:** postmortem interval, X-ray diffraction (XRD), attenuated total reflection Fourier transform infrared (ATR-FTIR) spectroscopy, teeth composition, teeth structure, forensic odontology

## Abstract

The postmortem interval (PMI) is difficult to estimate in later stages of decomposition. There is therefore a need to develop reliable methodologies to estimate late PMI. This study aims to assess whether there is a correlation between changes in the mineral composition of human teeth and the estimation of PMI. X-ray diffraction (XRD) and attenuated total reflection Fourier transform infrared (ATR-FTIR) spectroscopy techniques were performed to address this challenge. Forty healthy human teeth obtained from odontological clinics were stored at different times (0, 10, 25, 50 years; N = 10/group). XRD and ATR-FTIR parameters related to the structure and composition of teeth were studied. Our results showed that the crystallinity index, crystal size index, mineral-to-organic matrix ratio (M/M) and carbonate/phosphate ratio (C/P) had the strongest association with PMI. For larger PMIs, there was a significant increase in crystallinity, crystal size and M/M ratio, while the C/P ratio showed a specific decrease with increasing PMI. According to our results, the parameters of crystallinity, crystal size, M/M ratio and C/P ratio can be considered highly accurate in determining a PMI of 10 years of data; crystallinity and mineral maturity can be considered useful in determining a PMI of 25 years; and crystallinity and mineral maturity can be considered highly accurate in determining a PMI of 50 years. A particular XRD index was identified as the most suitable parameter to estimate PMI: crystallinity. The joint use of XRD and ATR-FTIR analyses could be a promising alternative for dating human teeth.

## 1. Introduction

Estimating the postmortem interval (PMI)—the time elapsed between the physiological death of an organism and its examination [1]—is one of the most relevant challenges in forensic anthropology and forensic medicine, and it has important legal implications. There are many approaches for estimating PMI in the early stages of decomposition [2]. In contrast, there are currently no reliable, accurate methods for determining PMI in the later stages of decomposition. The early postmortem period ranges from 3 to 72 h after death and is usually estimated using the classical postmortem changes (rigor mortis, livor mortis and algor mortis). The late postmortem interval begins with body tissue disintegration and is mainly described as decomposition or putrefaction, adipocere formation, mummification or skeletonization [3].

PMI estimation has been studied in a variety of samples at different stages of decomposition, including vitreous humor [4], blood [5], soft tissues [6] and skeletal remains [7]. A range of techniques have been applied to improve this estimate, such as thermogravimetry [8,9], micro-computed tomography [10], near-infrared spectroscopy [11], among others. New molecular approaches, such as proteomics [12], molecular [13,14] and microbiome techniques [15], are increasingly being considered for PMI estimation. X-ray diffraction (XRD) [16] and vibrational spectroscopy [7,17] are starting to be applied to the study of PMI. XRD in particular has been studied in human bone, with the finding that the degree of crystallinity of hydroxyapatite increases in the postmortem degradation process, showing a larger crystal size [16].

Individually, XRD and FTIR methods have several advantages: they are relatively easy to perform; they are fast, inexpensive techniques to study solids with a non-invasive method; and they only require a small amount of sample for detection (<1 mg) in a totally non-destructive manner [18]. This means they could be easily applied in forensic scenarios for estimating PMI. The late PMI has been studied using Fourier transform infrared (FTIR) spectroscopy in conjunction with chemometrics methods [7], using Raman spectroscopy and chemometrics [17], applying NIR spectroscopy (NIRONE^®^ Sensor X; Spectral Engines, 61449, Steinbach, Germany) [11], combining XRD and biochemical analyses in human bones [16] and using bacterial community succession in human bone [19], among others. In addition to the aforementioned techniques, other studies have focused on applying Fourier transform infrared spectroscopy (FTIR) in order to distinguish postmortem changes in various types of samples, such as human or animal tissue [20,21,22]. FTIR has also been used for postmortem studies in bone samples [7,23]. These works show that it is possible to distinguish between ancient and recent human bone by the crystallinity index and carbonate/phosphate index obtained from the FTIR spectra [18]. Other studies show that the spectra yielded by FTIR varied significantly in accordance with PMI, identifying the alteration of Amide I as the parameter that best estimated PMI in bone [7]. Furthermore, an increase in PMI leads to an increase in both the amount of Type-B carbonate and the carbonate/phosphate ratio but a decrease in the crystallinity index. The crystallinity index and carbonate ratio have therefore been identified as the most suitable FTIR and XRD indices for estimating PMI, especially in bones from females [23]. Despite extensive studies assessing compositional degradation in bone during PMI, there is a gap of knowledge regarding teeth, with very few studies on tooth composition using FTIR or XRD. In general, these few works focus on chemical differences in the composition of the different parts of the tooth in humans and animals [24,25,26,27,28]. However, teeth are a very valuable sample for PMI studies in forensic and anthropological practice, as they are highly resistant to postmortem degradation, putrefaction, aging and external environmental factors [29,30]. Moreover, they are commonly found in forensic, anthropological or archaeological settings [31,32]. Their high inorganic composition [33] makes teeth the hardest structures in the human body, and their location in the jawbone provides additional protection from putrefaction compared to bones [34]. Tooth degradation encompasses a series of structural and molecular changes, which lead to a decrease in complex organic molecules and a proportional increase in the inorganic matrix, which require in-depth study. However, to the best of our knowledge, there are no studies analyzing PMI by FTIR or XRD in human dental samples, meaning postmortem changes in tooth structure and composition remain poorly understood. This study therefore aims to assess whether there is a correlation between changes in the mineral composition of human teeth and the estimation of PMI by FTIR and XRD techniques. To this end, we employed XRD and Rietveld refinement for quantitative analysis of crystallographic parameters, along with attenuated total reflection Fourier transform infrared (ATR-FTIR) spectroscopy for quantification of relevant parameters that reflect the relative content of tooth compounds, such as phosphates (*_v_*_1*v*3_PO_4_^3−^), carbonates (*_v_*_2_CO_3_^2−^) and amides I (*_v_*C=O), in human teeth with different PMIs.

## 2. Results

### 2.1. Analysis of Crystallographic Indices in Teeth by XRD to Determine PMI

**Figure 1:**
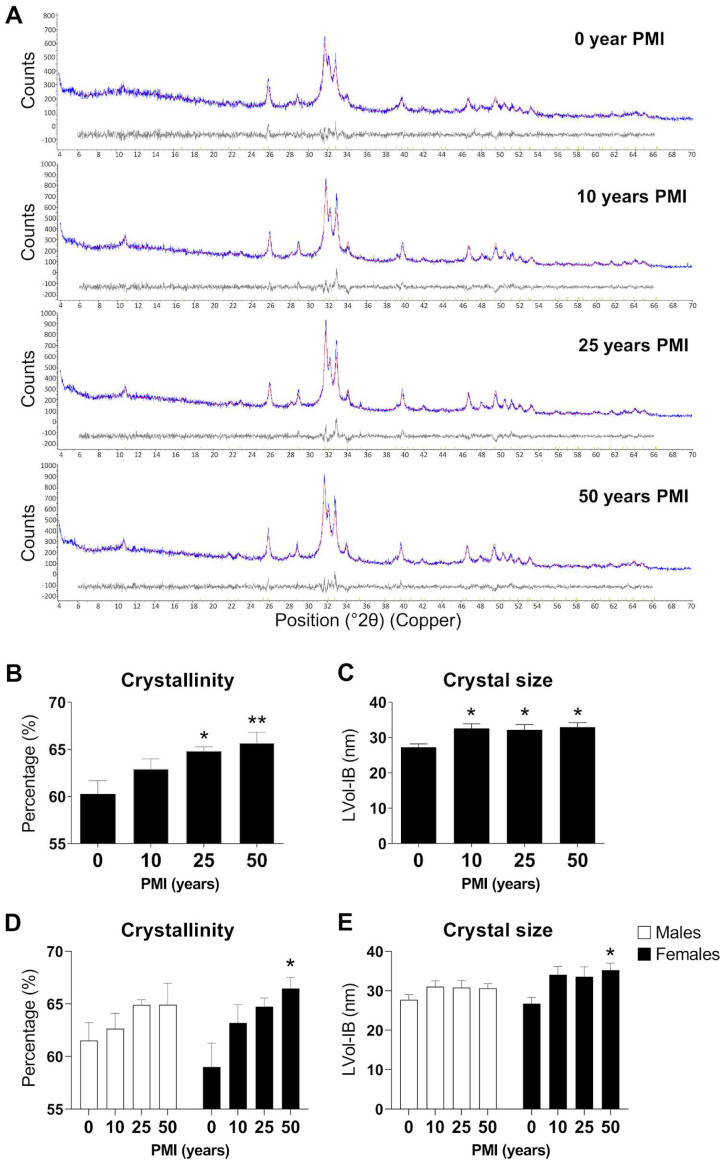
Analysis of crystallographic indices in teeth to determine the postmortem interval. (**A**): Representative XRD spectra for 0, 10, 25 and 50 years of PMI. (**B**): Crystallinity Index for 0, 10, 25 and 50 years of PMI. (**C**): Crystal Size for 0, 10, 25 and 50 years of PMI. (**D**): Crystallinity Index in males versus females for 0, 10, 25 and 50 years of PMI. (**E**): Crystal Size in males versus females for 0, 10, 25 and 50 years of PMI. Histograms represent means ± SD (*n* = 10). */** *p* < 0.05/0.01 versus control group.

The crystallographic indices of hydroxyapatite-(CaOH) found in the teeth were quantified by XRD and Rietveld refinement. Representative XRD spectra are shown in Figure 1A. We observed significant overall differences in the different PMI groups. Changes over the PMI were observed in two crystallographic indices: crystallinity (F_3,36_ = 5.120, *p* = 0.0047) and crystal size (F_3,36_ = 4.356, *p* = 0.0102). The crystallinity index showed an increase with increasing PMI, displaying significant differences between 0 years versus 25 (*p* < 0.05) and 50 (*p* < 0.01) years of PMI (Figure 1B). The crystal size index showed an increase with increasing PMI, with differences between 0 years compared to the rest of the groups (10, 25 and 50 years of PMI) (*p* < 0.01) (Figure 1C).

Analyzing crystallinity and crystal size by gender (Figure 1D) showed changes across PMI with significant overall differences in the different groups (F_3,32_ = 4.819, *p* = 0.0070 and F_3,32_ = 4.624, *p* = 0.0085, respectively). Although an interaction between gender and PMI was not found (Figure 1E), a specific increase in crystallinity index and crystal size was observed in the 50-year PMI group in females (*p* < 0.05).

### 2.2. Analysis of Tooth Composition by ATR-FTIR to Determine PMI

**Figure 2:**
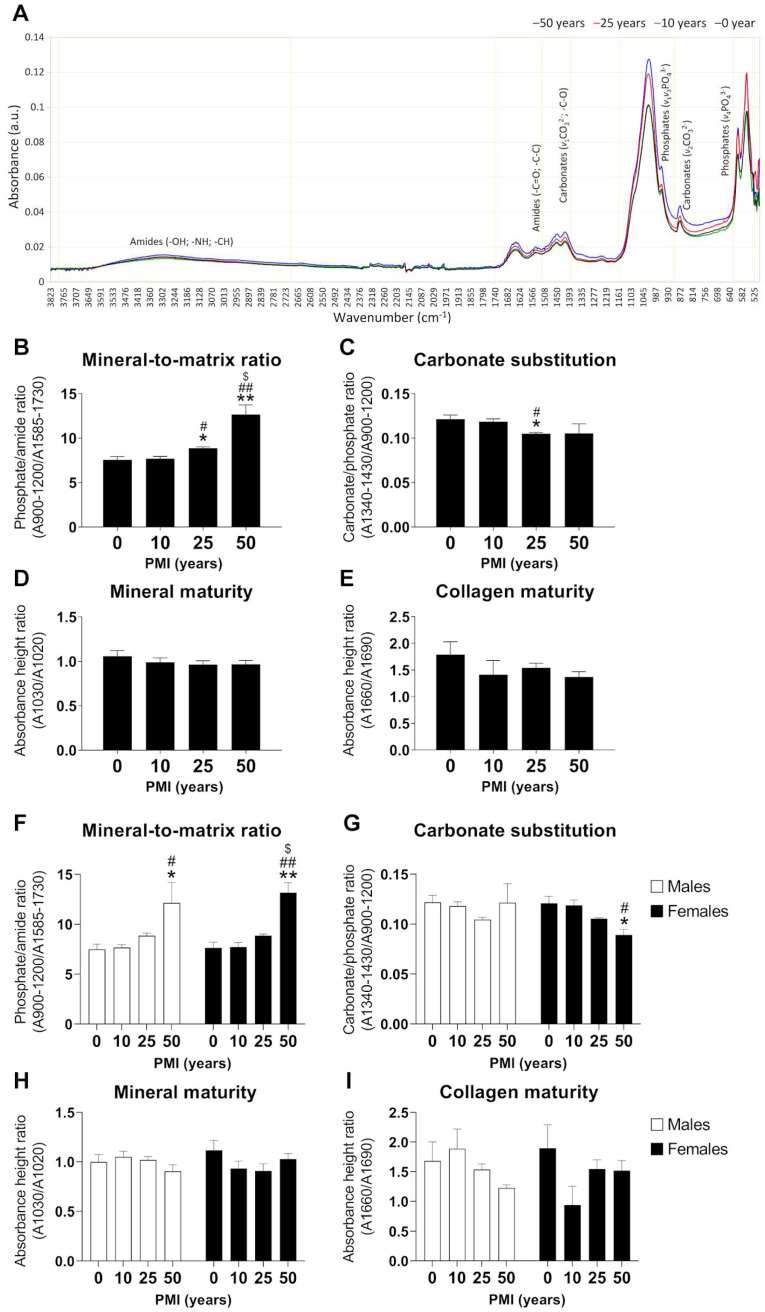
Analysis of tooth composition by ATR-FTIR to determine PMI. (**A**): Mean values of the ATR-FTIR spectra from each PMI group (0, 10, 25 and 50 years). (**B**): Effect of PMI on mineral-to-matrix ratio. (**C**): Effect of PMI on carbonate-to-phosphate ratio. (**D**): Effect of PMI on mineral maturity. (**E**): Effect of PMI on collagen maturity. (**F**): Effect of PMI on mineral-to-matrix ratio by gender. (**G**): Effect of PMI on carbonate-to-phosphate ratio by gender. (**H**): Effect of PMI on mineral maturity by gender. (**I**): Effect on collagen maturity by gender. Histograms represent means ± SD (*n* = 10). */** *p* < 0.05/0.01 versus control group; #/## *p* < 0.05/0.01 versus 10-year group; $ *p* < 0.05 versus 25-year group.

The relative contents of tooth compounds containing amides, carbonates and phosphates were identified by ATR-FTIR in all teeth of the different PMI groups. Figure 2A represents the mean values of the ATR-FTIR spectra from each PMI group. The relative content of tooth compounds was quantified to calculate relevant parameters related to tooth composition, such as mineral-to-matrix (M/M) ratio, carbonate-to-phosphate ratio (C/P ratio), mineral maturity and collagen maturity. An overall effect of PMI was found on the M/M ratio (W_3,17.99_ = 11.75, *p* = 0.0002) and C/P ratio (W_3,16.64_ = 7.71, *p* = 0.0019) (Figure 2B,C) but not on mineral maturity and collagen maturity (Figure 2D,E). The M/M ratio showed an increase with increasing PMI, exhibiting significant differences between 25 years versus 0 (*p* < 0.05) and 10 (*p* < 0.05) years of PMI, and between 50 years versus 0 (*p* < 0.01), 10 (*p* < 0.01) and 25 (*p* < 0.05) years of PMI (Figure 2B). The C/P ratio showed a decrease with increasing PMI, exhibiting significant differences between 25 years versus 0 (*p* < 0.05) and 10 (*p* < 0.05) years of PMI (Figure 2C).

Analyzing the M/M ratio, C/P ratio, mineral maturity and collagen maturity by gender (Figure 2F–I) revealed an overall effect of PMI on the M/M ratio (F_3,32_ = 14.83, *p* < 0.0001) (Figure 2F) but not on the C/P ratio, mineral maturity and collagen maturity (Figure 2G–I). Interestingly, an increase in the M/M ratio was found with increasing PMI, with significant differences between 50 years versus 0 (*p* < 0.05) and 10 (*p* < 0.05) years of PMI in males and between 50 years versus 0 (*p* < 0.01), 10 (*p* < 0.01) and 25 (*p* < 0.05) years of PMI in females (Figure 2F). In contrast, the C/P ratio showed a specific decrease with increasing PMI in females, with significant differences between 50 years versus 0 (*p* < 0.05) and 10 (*p* < 0.05) years of PMI (Figure 2G).

### 2.3. Correlation Analysis between PMI, Crystallographic Indices and Tooth Compounds

**Figure 3:**
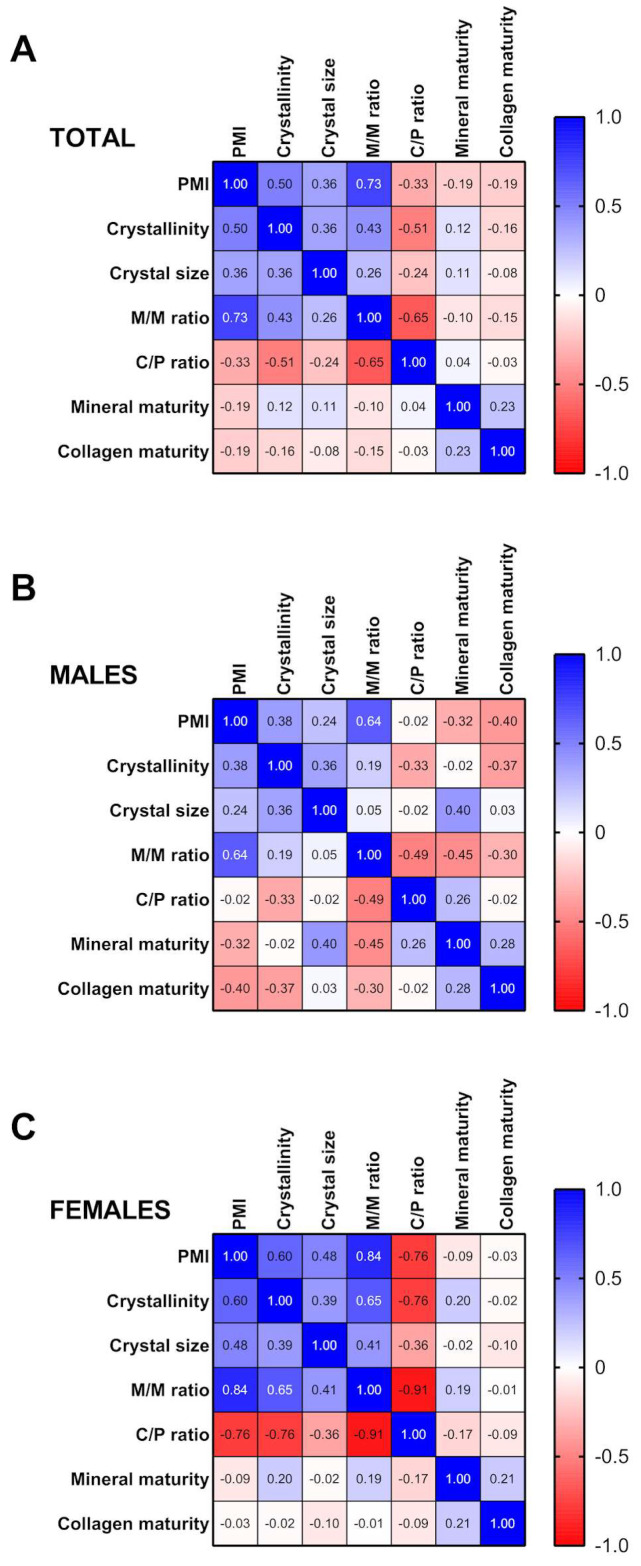
Correlation analysis between PMI, crystallographic indices and tooth compounds. (**A**): Correlation analysis between PMI, crystallographic indices and tooth compounds in the total sample. (**B**): Correlation analysis between PMI, crystallographic indices and tooth compounds in males. (**C**): Correlation analysis between PMI, crystallographic indices and tooth compounds in females.

The relationship between the different variables in all samples was explored using the Pearson r correlation. The analysis revealed that PMI correlated positively with crystallinity (rho = 0.50, *p* = 0.001), crystal size (rho = 0.36, *p* = 0.021) and the M/M ratio (rho = 0.73, *p* < 0.001). However, a negative correlation was found between PMI and the C/P ratio (rho = −0.33, *p* = 0.039), C/P ratio and crystallinity (rho = −0.51, *p* = 0.001) and C/P ratio and M/M ratio (rho = −0.65, *p* < 0.001) (Figure 3A and Table S1). A correlation analysis by gender revealed that PMI correlated positively with the M/M ratio (rho = 0.64, *p* = 0.002) in males (Figure 3B and Table S2). The M/M ratio also correlated negatively with C/P ratio (rho = −0.49, *p* = 0.028) and mineral maturity (rho = −0.45, *p* = 0.0047) in males (Figure 3B and Table S2). In females, PMI correlated positively with crystallinity (rho = 0.60, *p* = 0.005), crystal size (rho = 0.48, *p* = 0.034) and M/M ratio (rho = 0.84, *p* < 0.001) (Figure 3C and Table S3). In contrast, the C/P ratio correlated negatively with PMI (rho = −0.76, *p* < 0.001), crystallinity (rho = −0.76, *p* < 0.001) and M/M ratio (rho = −0.91, *p* < 0.001) in females (Figure 3C and Table S3).

### 2.4. Changes in Crystallographic Indices and Tooth Compound Parameters Explain PMI

After assessing the factor analysis and correlations, the selected model contained seven independent variables: the M/M ratio, C/P ratio, age of individuals, crystallinity, crystal size, mineral maturity and collagen maturity. The variances of the seven variables chosen were accounted for by three extracted components.

**Figure 4:**
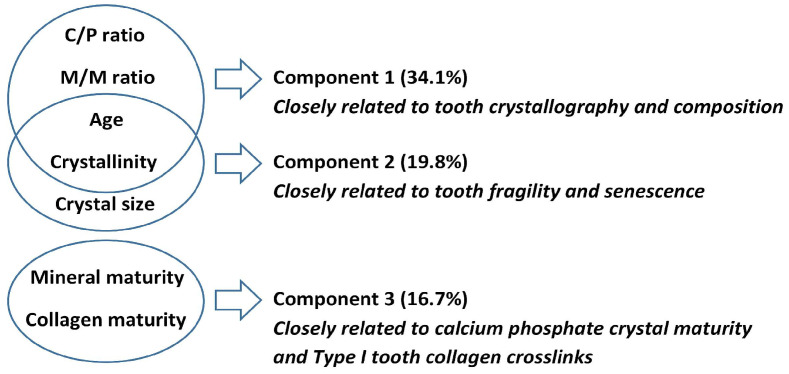
Component analysis explaining PMI in human teeth.

According to this model, the three components (factors) together explained 70.6% of the variance associated with PMI (0, 10, 25 and 50 years) in human teeth (Figure 4 and Table S4). The most influential factor (component 1) explained 34.1% of total variance. The C/P ratio, M/M ratio, age of individuals and crystallinity had high factor loadings (−0.789, 0.875, 0.666 and 0.578, respectively). Component 2 explained 19.8% of total variance, with age of individuals, crystallinity and crystal size having high factor loadings (−0.401, 0.557 and 0.840, respectively). Component 3 explained 16.7% of total variance. Mineral maturity and collagen maturity have high factor loadings (0.797 and 0.735, respectively).

### 2.5. Predicting PMI from Crystallographic Indices and Tooth Compound Parameters

**Figure 5:**
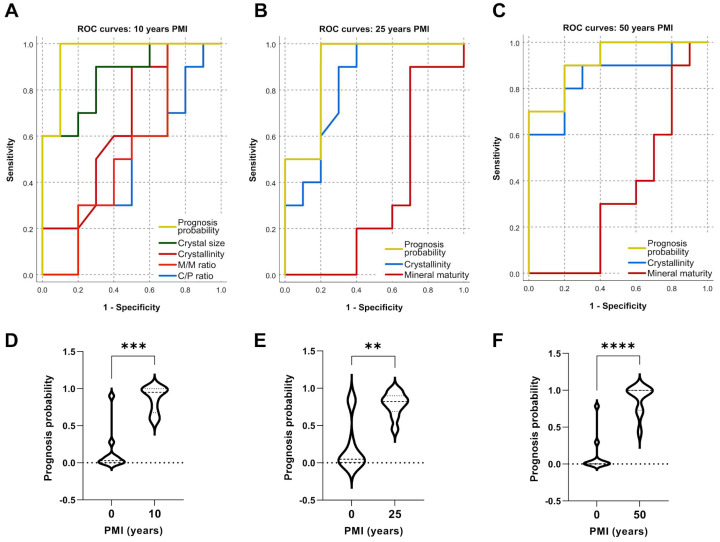
Receiver operating curve (ROC) and prognostic probability analysis predicting the postmortem interval (10, 25 and 50 years) in human teeth. (**A**): ROC Curve for 10 years of postmortem interval. (**B**): ROC Curve for 25 years of postmortem interval. (**C**): ROC Curve for 50 years of postmortem interval. (**D**): Prognosis probability for 10 years of postmortem interval. (**E**): Prognosis probability for 25 years of postmortem interval. (**F**): Prognosis probability for 50 years of postmortem interval. **/***/**** *p* < 0.01/0.001/0.0001 versus control group.

After evaluating binary logistic regression, the results showed that crystallinity, crystal size, M/M ratio and C/P ratio had the strongest association with PMI. The predicting variables selected for the 10 years of PMI were crystallinity, crystal size, M/M ratio and C/P ratio (Figure 5A). The overall success rate (percentage of correct predictions) for 10 years of PMI was 87% (ROC-AUC = 0.96, 95% CI = 0.87–1.04, Sensitivity = 0.9 and 1−Specificity = 0.1). The predicting variables selected for 25 years of PMI were crystallinity and mineral maturity (Figure 5B). The overall success rate for 25 years of PMI was 76% (ROC-AUC = 0.90, 95% CI = 0.75–1.04, Sensitivity = 0.9 and 1−Specificity = 0.2). The predicting variables selected for 50 years of PMI were crystallinity and mineral maturity (Figure 5C). The overall success rate for 50 years of PMI was 80% (ROC-AUC = 0.92, 95% CI = 0.80-1.03, Sensitivity = 0.9 and 1−Specificity = 0.2). Analysis of the predictive probabilities for teeth with 10 years (Figure 5D), 25 years (Figure 5E) and 50 years (Figure 5F) of PMI indicated that the respective means were significantly different compared to teeth with 0 years of PMI (10 years: U = 4, *p* = 0.0001; 25 years: U = 10, *p* = 0.0015; 50 years: U = 3, *p* < 0.0001). More information can be found in Table S5.

## 3. Discussion

This study addresses one of the main challenges in forensic science (accurately determining the PMI), carrying out an assessment in a non-common tissue (tooth) and combining two biochemical approaches—XRD and FTIR—for the first time.

To the best of our knowledge, this is the first study that uses XRD and ATR-FTIR jointly to predict PMI in human teeth. The results show that the combination of XRD and ATR-FTIR analysis is ideal for estimating late PMI based on time-dependent component changes in human teeth. A diagnostic test is considered “highly accurate” with an AUC value of >0.9, “useful for some purposes” with a value of 0.7–0.9 and “poor” with a value of 0.5–0.7 [35]. Our results show that the parameters of crystallinity, crystal size, M/M ratio and C/P ratio can be considered highly accurate in determining a PMI of 10 years of data with a lower 95% CI limit of AUC > 0.9 (Table S5). Applying the same strict statistical interpretation, crystallinity and mineral maturity can be considered useful in determining a PMI of 25 years with a lower 95% CI limit of AUC = 0.9 (Table S5). Crystallinity and mineral maturity can be considered highly accurate in determining a PMI of 50 years with a lower 95% CI limit of AUC > 0.9 (Table S5). This method can therefore be considered highly accurate in estimating PMI at 10 and 50 years of data and useful in estimating PMI at 25 years of data in forensic and anthropological cases. In our results, the parameters of XRD and ATR-FTIR accurately discriminated between PMI times in sample teeth, with crystallinity being the most useful due to its applicability in all PMI studied.

There is a close association between the mineral and the organic matrix of the mineralized samples, leading to a low degree of crystallinity of the hydroxyapatite [15]. Molecular mechanisms during the fossilization process, such as a decrease in the organic matter, results in increased crystallinity [15]. Therefore, it is to be expected that as a mineralized sample begins to lose organic matter in its degradation process, the degree of crystallinity of the hydroxyapatite increases, showing a larger crystal size and, therefore, changes in XRD and FTIR peaks. In this process of loss of organic matter during postmortem degradation, molecular mechanisms play a key role in the relationship between collagen structure and crystallinity. This relationship will produce changes in the strength and fragility of the mineralized samples over time [15,18]. Our results clearly show that the crystallinity index, crystal size and M/M ratio increase significantly with increasing postmortem interval in sample teeth, displaying analogous results to those of XRD applied in bone samples [15,18]. This may be due to the fact that bone begins to lose organic matter while crystallinity increases during the postmortem degradation process, showing a more ordered, larger size of crystals [15,20,36]. Our results would appear to indicate that this same molecular mechanism could occur in the human tooth. In contrast, a decrease in the C/P ratio with increasing PMI in human teeth was observed, suggesting different results from other authors [23]. The explanation for these differences may be due to the fact that the composition of teeth is more heterogeneous than that of bone due to the structure of the enamel, dentin and cementum that compose the teeth [37]. In this sense, teeth and bone tissues are composed of the same elements (water, organic matter and a mineral phase) but in different proportions. The mineral matrix of bone and teeth is mainly composed of hydroxyapatite crystals. These crystals differ in size and quantity for each mineralized tissue (bone and teeth). Moreover, the tooth contains enamel, which has a very small amount of organic matrix (>1% weight (wt)) [26]. Therefore, the differences presented can be attributed to the proportional variation of the components of the two mineralized tissues and the enamel structure.

Age has implications for the characteristics of the teeth matrix [31]. For example, the mineral content of dentin increases with age and, in turn, affects the organic content of dentin [31]. In this regard, our results showed that age contributes to the PMI variance in Component 1 (closely related to tooth crystallography and composition) and Component 2 (closely related to tooth fragility and senescence). Regarding the effects of sex on PMI, we observed that an increase in PMI is associated with an increase in crystallinity and crystal size in females but not in males. The mean age of the females in our study is over 60 years; this circumstance may indicate that hormonal changes after menopause could influence the results obtained for an estimate of the PMI.

The assessment of taphonomic changes in a body is essential in a forensic anthropology analysis. Teeth could be affected by various taphonomic and diagenetic processes, which may modify the structure and composition of teeth biomaterials [37]. In addition, several factors can affect the decomposition of a body by accelerating or suppressing the periods of putrefaction, such as the flora, fauna, type of burial, ambient temperature, soil characteristics, humidity, rainfall, age, sex, etc. [23]. As an example, during the burial period, accumulation of carbonate may occur over time in mineralized tissues, depending on the soil conditions at the burial site [23]. However, teeth have a unique composition and are more protected than bones from postmortem degradation, aging and external environmental factors due to their higher inorganic composition and their inclusion in the jawbone [29,34]. Our study was performed on dental samples and under in vitro and controlled conditions, so the environmental effects of real forensic cases could not be explored. This research is a preliminary investigation to evaluate the application of XRD and FTIR conjointly to estimate PMI, but, considering our results, it could be a promising alternative in the future for use in the dating of human teeth.

Our study has strengths and limitations. The principal strength is the novelty of the study, it being the first one to provide a highly accurate estimation method for PMI by combining XRD and FTIR-ATR on human teeth samples stored at 10, 25 and 50 years of PMI, all using one of the hardest tissues in the human body—the tooth—thus allowing application in severely decomposed bodies. Our study has several limitations. The PMI was researched in a controlled laboratory environment, and no account was taken of influential factors, such as soil, temperature, environmental conditions, among others. In addition, the sample size of the study was small, so more studies should be replicated to be representative of the general population. Further studies are needed in order to analyze the effect of other factors on PMI in teeth (e.g., gender, age, tooth type, healthy and unhealthy tooth), since differences in the chemical composition can occur in the same teeth or between different individuals.

## 4. Materials and Methods

This study was approved by the Research Ethics Committee of Málaga Province (approval reference: ODONTAGING-2021; approval date: 16 December 2021) and conducted in accordance with the Declaration of Helsinki and national data protection legislation. Informed consent was obtained from all subjects.

### 4.1. Samples

A total of 40 healthy human teeth (molars and premolars) were obtained from adult patients (20 females and 20 males) aged between 29 and 82 years (mean of 60 ± 11.54 years) in public and private dental clinics in Granada, Málaga and Cádiz (Spain). All the teeth studied were extracted for valid clinical reasons (periodontal disease or orthodontic treatment) and were free of caries, fillings and fractures (Table S6).

After extraction, the teeth were washed with distilled water, and their external surfaces were cleaned with curettes to remove any extraneous material. The teeth were then stored under controlled conditions of 21 °C and 65% humidity for 0, 10, 25 and 50 years.

### 4.2. Sample Preparation

The teeth were pulverized in liquid nitrogen using a 6770 Freezer Mill (SPEX CertiPrep FreezerMill, Stanmore, London, UK). The resulting powder was collected and stored in a −80 °C freezer until XRD and ATR-FTIR analysis.

### 4.3. X-ray Powder Diffraction

The teeth were analyzed (~100 mg) using an Empyrean Malvern Panalytical automated X-ray diffractometer (Malvern Panalytical, Malvern, UK) and Rietveld refinement [38,39,40,41,42]. The sample crystallinity and crystallite size patterns were collected with step size 0.017° (2θ) and 300 sec/step using Cu-Kα (λ = 1.540598 Å) radiation from a tube operated at an accelerating voltage of 45 kV and a current of 35 mA. The (002) peak was baselined from 4° to 80° (2θ) for 30 min and fitted with a Lorentzian curve to determine peak broadening as a function of its full width at half maximum. Identification of the amorphous phase and pure crystalline material was performed with reference to an external standard and the database provided by the International Center for Diffraction Data (Powder Diffraction File no. 84-1998), Inorganic Crystal Structure Database and Crystallography Open Database (COD no. 9010050; RRID:SCR_005874). Sample crystallinity (the degree of order in a solid) is defined as the ratio of the enthalpy difference between the pure amorphous phase and the sample enthalpy over the difference of the pure amorphous and the pure crystalline material (external standard). Crystallinity percentage is calculated with the following formula: (total area of crystalline peaks) x100/(total area of crystalline and amorphous peaks). The Scherrer equation (Dv = K x λ/β002 x cosθ) and Williamson–Hall method were used to calculate crystallite size (LVol-IB, nm), where Dv is the volume weighted crystallite size, K is the Scherrer constant with a value of 1, λ is the X-ray wavelength used, and β002 is the integral breadth of the (002) reflection or length of the apatite crystals along the C-axis. The R-Bragg factor, cell volume, crystal linear absorbance coefficient (1/cm) and crystal density (g/cm^3^) were also checked. Three patterns were performed, obtaining a mean pattern for each sample.

### 4.4. ATR-FTIR Spectroscopy

Infrared (IR) analysis of each tooth (~100 mg) was carried out in a Bruker Vertex 70 Fourier Transform (FT)-IR spectrophotometer (Bruker Corporation, Billerica, MA, USA). Attenuated total reflectance (ATR) was used with a Golden Gate System of Individual Reflection [42,43,44,45]. The internal reflection element was ZnSe (20,000–500 cm^−1^). For acquisition of the spectra, a standard spectral resolution of 4 cm^−1^ in the spectral range of 4000–500 cm^−1^ was used, along with 64 accumulations per sample. The background spectrum in all cases was the air. For analysis of raw spectra, the *_v_*_1*v*3_PO_4_^3−^ bands were baselined from 1200 to 900 cm^−1^, the *_v_*_2_CO_3_^2−^ band from 890 to 850 cm^−1^ and the amide I band from 1730 to 1585 cm^−1^. Spectral analysis was performed in triplicate, and a mean spectrum was obtained for each sample. The position, height and area under the curves (baseline correction) were measured after curve-fitting every individual (not smoothing) spectrum.

The following parameters reflecting the compositional properties of dental samples were calculated [26,42,46,47,48]: (1) mineral-to-organic matrix (M/M) ratio, an index of mineral content that characterizes the relative amount of phosphate per amount of collagen present, calculated by the ratio of the integrated areas of the respective raw peaks of *_v_*_1*v*3_PO_4_^3−^ (1200–900 cm^−1^) and amide I (1730–1585 cm^−1^); (2) carbonate-to-phosphate ratio or carbonate-to-mineral ratio (C/P ratio), an index of phosphate-to-carbonate-substituted apatites that characterizes the degree of carbonate substitutes in the mineral lattice, calculated by the ratio of the integrated areas of the respective raw peaks of *_v_*_2_CO_3_^2−^ (890–850 cm^−1^) and *_v_*_1*v*3_PO_4_^3−^ (1200–900 cm^−1^); (3) mineral crystallinity or maturity (1030/1020 cm^−1^ intensity ratio), a degree of order in a solid, which is related to the size and perfection of crystals; and (4) collagen maturity (1660/1690 cm^−1^ intensity ratio), an index related to the ratio of mature, non-reducible collagen crosslinks to immature, reducible collagen crosslinks. The second derivatives of the raw data from the ATR-FTIR spectra were applied to determine specific peaks at ~1030, ~1020, ~1660 and ~1690 cm^−1^ and improve the accuracy of the quantification of mineral maturity and collagen crosslink ratio.

### 4.5. Statistical Analysis

GraphPad Prism 9.0 and IBM SPSS Statistics 26.0 were used for statistical analysis. All data are represented as the mean ± standard error of the mean (SEM) of 10 determinations per experimental group (*n* = 10). The normal (Gaussian) distribution of the variables was assessed by the Dallal–Wilkinson–Lilliefor corrected Kolmogorov–Smirnov test. Most variables met the assumption of Gaussian distribution (*p* > 0.1), and parametric statistics were applied. Otherwise, statistical analyses were performed using non-parametric Mann–Whitney U-test for comparison between two groups. Bartlett’s test was performed to assess equal variances across groups. Firstly, the comparison of quantitative variables between PMI groups that assumed equal standard deviation (SD) were performed using an ANCOVA test, followed by Tukey’s test for multiple comparisons. Quantitative variables that did not assume equal SD were analyzed using Welch’s ANOVA test, followed by Dunnett’s T3 test (*n* < 50/group) for multiple comparisons. Age was controlled, being entered as a covariate. Secondly, a two-way ANOVA test was carried out to assess the effects of PMI and gender as the main factors and the interaction between them, followed by Tukey’s test for multiple comparisons. Thirdly, a Pearson correlation test was performed to analyze the relationship between variables. Fourthly, principal component analysis with orthogonal (varimax) rotation between variables was undertaken to determine the components that account for PMI. Only variables with a factor loading of at least 0.4 (sharing at least 10% of the variance with a factor) were considered high enough for interpretation. Finally, the backward method for binary logistic regression and receiver operating characteristic (ROC) analysis was used to determine the predictability of each PMI. These steps were taken to obtain all possible combinations of the exploratory variables and to calculate the highest areas under the ROC curves (AUCs) and the overall success rates (percentage of correct predictions) of the resulting model with this combination (i.e., the one with the greatest discrimination power). A *p*-value below 0.05 was considered significant.

## 5. Conclusions

The results show that the combination of FTIR-ATR analysis and XRD is ideal for estimating late PMI based on time-dependent component changes in human teeth. According to our results, PMI has a strong association with crystallinity, crystal size, M/M ratio and C/P ratio, and the crystallographic parameter that best predicts PMI is crystallinity. Molecular mechanisms underlying the changes observed in crystallinity are related with the loss of organic matter in the PMI. In the overall analysis of our data, the combination of XRD and ATR-FTIR analyses could be a promising alternative for use in the dating of human teeth. These results may help better understand the molecular mechanisms of the degradation of human teeth and provide a basis for future practical research.

## Data Availability

The data that support the findings of this study are available on reasonable request from the corresponding author.

## Supplementary Materials

The supporting information can be downloaded at: https://www.mdpi.com/article/10.3390/ijms24076793/s1.
