# Supplementary material for: Partners in Postmortem Interval Estimation: X-ray Diffraction and Fourier Transform Spectroscopy
Source: Int J Mol Sci. 2023 Apr 5;24(7):6793. doi: 10.3390/ijms24076793 (PMC10094861; doi:10.3390/ijms24076793)
Supplement: Supplementary file 1 [file ijms-24-06793-s001.zip › ijms-2299138-supplementary.pdf]

Supplementary Material

Partners in Postmortem Interval Estimation: X-ray Diffraction and Fourier Transform Spectroscopy

Leticia Rubio [1,2,3,4,*], Juan Suárez [1,2,3,4], Stella Martin-de-las-Heras [2,3,*] and Sara C. Zapico [4,5]

1 Fulbright Visiting Scholar Program. Department of Chemistry and Environmental Sciences, New Jersey Institute of Technology, Tiernan Hall 365, Newark, NJ 07102, USA; juan.suarez@uma.es

2 Departamento de Anatomía Humana, Medicina Legal e Historia de la Ciencia, Facultad de Medicina, Universidad de Málaga, 29071 Málaga, Spain

3 Instituto de Investigación Biomédica de Málaga-IBIMA, 29590 Málaga, Spain

4 Department of Chemistry and Environmental Sciences, New Jersey Institute of Technology, Tiernan Hall 365, Newark, NJ 07102, USA; sc338@njit.edu

5 Department of Anthropology, NMNH-MRC 112, Smithsonian Institution, Washington, DC 20560, USA

* Correspondence: lorubio@uma.es (L.R.); smdelasheras@uma.es (S.M.-d.-l.-H.)

Table S1. Correlation matrix among crystallographic indexes and compositional parameters in human teeth.

Variables	Age	Crystallinity	Crystal size	M/M ratio	C/P ratio	Mineral maturity	Collagen maturity
PMI	0.407<br>***0.009***	0.500<br>***0.001***	0.363<br>***0.021***	0.732<br>***<0.001***	-0.328<br>***0.039***	-0.190<br>ns	-0.194<br>ns
Crystallinity			0.360<br>***0.022***	0.426<br>***0.006***	-0.505<br>***0.001***	0.117<br>ns	-0.159<br>ns
Crystal size				0.257<br>ns	-0.236<br>ns	0.112<br>ns	-0.076<br>ns
M/M ratio					-0.654<br>***<0.001***	-0.099<br>ns	-0.145<br>ns
C/P ratio						0.040<br>ns	-0.033<br>ns
Mineral maturity							0.232<br>ns

Collagen maturity							

Correlation matrix showing correlation coefficients between a single variable and every other variable. *p*-values are also shown (significance in bold).

Table S2. Correlation matrix among crystallographic indexes and compositional parameters in human male teeth.

Variables	Age	Crystallinity	Crystal size	M/M ratio	C/P ratio	Mineral maturity	Collagen maturity
PMI	0.615<br>***0.004***	0.382<br>ns	0.241<br>ns	0.641<br>***0.002***	-0.020<br>ns	-0.324<br>ns	-0.401<br>ns
Crystallinity			0.362<br>ns	0.189<br>ns	-0.333<br>ns	-0.016<br>ns	-0.373<br>ns
Crystal size				0.049<br>ns	-0.021<br>ns	0.401<br>ns	-0.030<br>ns
M/M ratio					-0.492<br>***0.028***	-0.449<br>***0.047***	-0.295<br>ns
C/P ratio						0.257<br>ns	-0.020<br>ns
Mineral maturity							0.277<br>ns
Collagen maturity							

Correlation matrix showing correlation coefficients between a single variable and every other variable. *p*-values are also shown (significance in bold).

Table S3. Correlation matrix among crystallographic indexes and compositional parameters in human female teeth.

Variables	Age	Crystallinity	Crystal size	M/M ratio	C/P ratio	Mineral maturity	Collagen maturity
PMI	0.302<br>ns	0.601<br>***0.005***	0.475<br>***0.034***	0.837<br>***<0.001***	-0.763<br>***<0.001***	-0.093<br>ns	-0.029<br>ns
Crystallinity			0.392<br>ns	0.651<br>***0.002***	-0.757<br>***<0.001***	0.199<br>ns	-0.021<br>ns
Crystal size				0.406<br>ns	-0.363<br>ns	-0.018<br>ns	-0.097<br>ns
M/M ratio					-0.909<br>***<0.001***	0.186<br>ns	-0.005<br>ns
C/P ratio						-0.168<br>ns	-0.091<br>ns
Mineral maturity							0.207<br>ns

Collagen maturity	

Correlation matrix showing correlation coefficients between a single variable and every other variable. *p*-values are also shown (significance in bold).

Table S4. Crystallographic indexes and compositional parameters with high loadings in extracted components that contribute towards PMI (0-10-25-50 years) in human teeth.

**Variables**	**Component 1 (34.1%)**	**Component 2 (19.8%)**	**Component 3 (16.7%)**
M/M ratio	0.875	-	-
C/P ratio	-0.789	-	-
Age	0.666	-0.401	-
Crystallinity	0.578	0.557	-
Crystal size	-	0.840	-
Mineral maturity	-	-	0.797
Collagen maturity	-	-	0.735

Rotated orthogonal (varimax) component matrix from principal component analysis. A factor loading >0.4 was considered high enough for interpretation (shown). Components together explain 70.5% of the variance that contribute towards PMI. $\chi 2 = 34.64$, $p < 0.001$.

Table S5. Crystallographic indexes and compositional parameters as predictors for PMI in human teeth.

**Variables**	**0-10 PMI**			**0-25 PMI**			**0-50 PMI**		
	B	Wald χ^2	*p*	B	Wald χ^2	*p*	B	Wald χ^2	*p*
(Intercept)	133.908	1.393	*0.238*	-41.077	2.840	*0.092*	-20.919	4.077	***0.043***
Crystallinity	0.569	2.420	*0.120*	0.817	3.893	***0.048***	0.457	5.735	0.017
Crystal size	1.403	3.191	*0.074*	-	-	-	-	-	-
M/M ratio	-14.862	2.604	*0.107*	-	-	-	-	-	-
C/P ratio	-811.79	2.084	*0.149*	-	-	-	-	-	-
Mineral maturity	-	-	-	-10.658	2.865	*0.091*	-7.901	2.834	0.092
Collagen maturity	-	-	-	-	-	-	-	-	-
Age	-	-	-	-	-	-	-	-	-
Backward step	3			6			6		
Cox & Snell R^2		0.604			0.508			0.445	
Nagelkerke R^2		0.806			0.677			0.593	
Sensitivity		0.9			0.9			0.9	
1-Specificity		0.1			0.2			0.2	

95% CI	0.875-1.045	0.755-1.044	0.803-1.037
AUC	0.96	0.90	0.92
Overall rate	87%	76%	80%

Logistic regression (backward method). Variables entered on step 1: age, crystallinity, crystal size, mineral-to-matrix (M/M) ratio, carbonate substitution (C/P ratio), mineral maturity and collagen maturity. A stepping method criteria of 0.05 (entry), 0.2 (removal) and 0.5 (cutoff) were considered. *p*-values are also shown (significance in bold).

Table S6. Characteristics of the samples.

SEX	AGE	TEETH*	PMI (years)
Male	55	28	0
Female	59	44	0
Male	50	44	0
Male	57	27	0
Female	77	45	0
Male	60	38	0
Male	50	25	0
Female	74	44	0
Female	57	38	0
Female	80	44	0
Male	51	37	10
Female	53	28	10

Male	55	28	10
Female	40	36	10
Male	39	38	10
Female	53	25	10
Female	42	44	10
Male	51	14	10
Female	29	28	10
Male	74	25	10
Male	58	25	25
Female	59	24	25
Male	60	45	25
Male	62	45	25
Female	56	45	25
Female	64	14	25
Male	56	45	25
Male	65	25	25
Female	63	34	25
Female	60	44	25
Male	68	17	50
Female	62	27	50
Female	82	45	50
Male	68	44	50
Male	73	44	50
Female	68	44	50
Female	82	34	50
Male	65	35	50
Male	61	35	50
Female	62	27	50

*Teeth are listed according to the FDI nomenclature . PMI: Postmortem Interval
